# Characterization of Composite Agarose–Collagen Hydrogels for Chondrocyte Culture

**DOI:** 10.1007/s10439-024-03613-x

**Published:** 2024-09-14

**Authors:** Clarisse Zigan, Claudia Benito Alston, Aritra Chatterjee, Luis Solorio, Deva D. Chan

**Affiliations:** 1 Weldon School of Biomedical Engineering, Purdue University, West Lafayette, IN USA; 2 Department of Mechanical Engineering, Birla Institute of Technology and Science, Pilani, Hyderabad Campus, Hyderabad, Telangana India; 3 School of Mechanical Engineering, Purdue University, West Lafayette, IN USA

**Keywords:** Agarose, Collagen, Hydrogel, Biomaterials, Chondrocyte, Extracellular matrix

## Abstract

To elucidate the mechanisms of cellular mechanotransduction, it is necessary to employ biomaterials that effectively merge biofunctionality with appropriate mechanical characteristics. Agarose and collagen separately are common biopolymers used in cartilage mechanobiology and mechanotransduction studies but lack features that make them ideal for functional engineered cartilage. In this study, agarose is blended with collagen type I to create hydrogels with final concentrations of 4% w/v or 2% w/v agarose with 2 mg/mL collagen. We hypothesized that the addition of collagen into a high-concentration agarose hydrogel does not diminish mechanical properties. Acellular and cell-laden studies were completed to assess rheologic and compressive properties, contraction, and structural homogeneity in addition to cell proliferation and sulfated glycosaminoglycan production. Over 21 days in culture, cellular 4% agarose–2 mg/mL collagen I hydrogels seeded with primary murine chondrocytes displayed structural and bulk mechanical behaviors that did not significantly alter from 4% agarose-only hydrogels, cell proliferation, and continual glycosaminoglycan production, indicating promise toward the development of an effective hydrogel for chondrocyte mechanotransduction and mechanobiology studies.

## Introduction

Cartilage mechanobiology and tissue engineering fields have both evolved using strategies employed to repair or regenerate damaged cartilage using a combination of biomaterials, cells, and stimulation. It is well accepted that applying mechanical forces to engineered cartilage constructs can help mimic the natural environment and stimulate chondrocytes to produce extracellular matrix (ECM) components and maintain tissue integrity to enhance the formation of functional cartilage tissue [1–3]. However, the underlying mechanisms that are employed during these processes are poorly understood [4, 5]. Many studies focus primarily on the effects of loading conditions applied to cartilage constructs [6–10]. However, another key factor to consider is construct composition, since administered mechanical forces can be influenced by exogenous physical and chemical cues of the 3-dimensional matrix that envelops the cell [11]. Cartilage-like constructs are often formed of polymeric hydrogels [12, 13].

Agarose is a common natural polymer used for cartilage mechanobiology studies due to its easily tunable mechanical stiffness. However, prior chondrocyte biology studies use low (2–3% v/v) concentration agarose-based hydrogels [14–18], which do not achieve cartilage-like mechanical properties as their corresponding moduli range from 13 to 18 kPa. Zignego et al., however, found that higher concentration 4.5% w/v agarose hydrogels achieve stiffness levels within ranges found in the native pericellular matrix (PCM; 20–200 kPa [19, 20]) while still maintaining chondrocyte viability, highlighting potential of high stiffness microenvironments to better match cartilage properties for subsequent mechanotransduction studies [21]. However, without modification, agarose lacks integrin binding motifs that are critical in the mediation of cell–ECM interactions.

Collagen I is another natural polymer common in chondrocyte studies [22–24]. Unlike agarose, collagen offers ample binding motifs [25], to promote cell adhesion and migration. Collagen hydrogels have a large water content leading to flexibility similar to natural tissue, exhibit biocompatibility and biodegradation abilities, and can be mechanical tuned through physical or chemical crosslinking [12]. While type II collagen is the most abundant extracellular molecule in cartilage, providing foundation for the use of collagen hydrogels, in vitro studies generally use type I collagen hydrogels since they demonstrate superior mechanical qualities compared to type II collagen. Prior collagen hydrogel studies used concentrations between 0.5 and 3 mg/mL [22, 26, 27] and even up to 7.5 mg/mL [28, 29]. Low-concentration collagen hydrogels (< 3 mg/mL) poorly maintain their 3-dimensional structure in extended studies [22] and have elastic moduli of 5–22 Pa [22, 28, 30], well below that of native cartilage. Although collagen concentration can be increased for greater strength, high-concentration collagen (5 mg/mL) has been shown to negatively impact the bioactivity of cells embedded within collagen–agarose hydrogels [31].

Composite hydrogels combine the favorable properties of each individual polymer, enabling tunable mechanical and structural properties and leading to more ECM-like interactions that promote cues for proliferation, differentiation, and matrix production. Few groups have studied the interactions between agarose and collagen biomaterials and their influence on cells [31–36]. Cambria et al. assessed the impact of low-concentration agarose blended with collagen on nucleus pulposus cells, finding composite hydrogels to outperform agarose-only hydrogels in terms of cell adhesion and proliferation, likely attributable to the binding motifs that exist within the collagen component [32]. Quarta et al. cultured breast cancer cell lines in agarose–collagen hydrogels to assess the mechanical properties and potential cytotoxicity as agarose content was increased, of which at the low concentrations assessed, while there was a change in mechanical behavior, no cytotoxicity was observed [33]. Ulrich et al. used another cancer cell line to assess how an increase in hydrogel stiffness would alter cell motility and therefore the potential influence of agarose on collagen deformation and remodeling [34]. While the group’s work with mass spectrometry and scanning electron microscopy fails to indicate agarose induces collagen ligand alterations, microscopy images do suggest agarose inhibits cell-directed assembly of large collagen bundles, also influencing cell spreading and motility [34]. While these studies demonstrate applications of agarose–collagen hydrogels and their compositional influence on mechanics, cell viability, and remodeling, study of the response of chondrocytes to this environment is critical to the application of such hydrogels to the study of cartilage mechanobiology.

In this study, we aimed to develop an agarose–collagen composite hydrogel that combines the mechanical properties of agarose with the biofunctionality of collagen to mimic native articular cartilage tissue and enable future chondrocyte mechanobiology studies. We hypothesized that the addition of collagen to agarose hydrogels will promote enhanced cell proliferation and subsequent matrix deposition as compared to agarose-only hydrogels without any significant compromise to material properties. Comparing against agarose- and collagen-only hydrogels, we evaluated agarose–collagen composite hydrogel mechanical properties and structural homogeneity alongside their effect on chondrocyte viability, proliferation, and sulfated glycosaminoglycan (sGAG) production.

## Materials and Methods

Experiments conducted in this study were divided into two main categories: (1) acellular and (2) cell laden. All hydrogel formulations were otherwise prepared in the same fashion. The aim of acellular experiments was to elucidate the innate structure–function relationships between the hydrogel formulation and the resulting bulk mechanics and structural homogeneity. Cell-laden hydrogel experiments were then used to assess chondrocyte morphology, proliferation rates, and sGAG production.

### Hydrogel Preparation

To prepare agarose hydrogels, low-gelling temperature type VII-A agarose (A0701, Sigma Aldrich) was dissolved in 1 × PBS solution and autoclaved (chamber temperature of 120 °C and sterilization time set to 15 min). For cell-based experiments, the solution was cooled to 40 °C prior to adding chondrocytes and casting in the mold.

Collagen I hydrogels were prepared by chilling and mixing rat tail collagen I (RatCol®; 5153, Advanced BioMatrix) with its associated neutralizing solution in accordance with the manufacturer protocol to obtain a 4 mg/mL solution.

Two composite hydrogel formulations were studied. The first composite consisted of 4% w/v agarose with 2 mg/mL collagen I. The second composite consisted of a 1:1 ratio of the aforementioned agarose-only and collagen-only formulations, leading to 2% w/v agarose with 2 mg/mL collagen I concentration. Composite hydrogels were manually mixed via pipetting while maintained in a ~ 40 °C water bath to prevent collagen denaturation and premature agarose gelation.

Positive displacement pipette tips were used to aliquot 150 μL volumes of each hydrogel solution into custom (3 mm tall, 6 mm inner diameter) PDMS-based molds (00–30, Ecoflex).

### Cell Encapsulation

To obtain primary chondrocytes, seven C57BL/6J mice (5 days old) were humanely euthanized under institutional approval (IACUC protocol 2104002138) to isolate neonatal cartilage of the proximal and distal femur and the proximal tibia under sterile conditions [37]. Tissues were digested in 3 mg/mL type II collagenase (17101-015, Gibco) reconstituted in culture media for 1 h at 37 °C followed by digestion in 0.5 mg/mL type II collagenase overnight at 37 °C. Tissue was agitated to dissociate residual tissue pieces, and the entire solution was filtered through a 40-μm cell strainer into a 50-mL conical tube. Sterile 1 × PBS was added until a 40 mL total volume was reached, at which point the solution was centrifuged at 1000 rpm for 10 min. The supernatant was aspirated, and the cell pellet resuspended in 30 mL of sterile 1 × PBS to be re-centrifuged at 1000 rpm for 10 min. The supernatant was again aspirated, and the pellet resuspended in complete growth media and counted.

Chondrocytes were resuspended into hydrogels at concentrations of 1 × 10^6^ cells/mL prior to full hydrogel gelation. Low seeding densities in the range of 0.4–6 × 10^6^ cells/mL exhibit proliferation, preserved chondrogenic markers, and reduced dedifferentiation [38–40]. Hydrogels with and without cells were allowed to solidify at room temperature for 10 min, followed by the addition of 0.5 mL complete culture media [phenol-free 4.5 g/L glucose Dulbecco’s modified Eagle medium (DMEM; 31053-036, Gibco) supplemented with L-glutamate (AAJ6057322, Gibco) and sodium pyruvate (11360-070, Gibco) and completed with 10% fetal bovine serum (FBS; 12676029, Corning) and 1% penicillin–streptomycin (15140-122, Gibco)]. After the addition of media, collagen fibrillogenesis was allowed to complete at 37 °C for 30 min, the amount of time needed to detect collagen fibrils after induction of fibrillogenesis [31, 32, 41]. Hydrogels were cultured in a 37 °C, 5% CO_2_ incubator in 48-well plates for up to 21 days. Culture medium was changed every 2–3 days.

### Rheological Characterization of Acellular Hydrogels

Following recommendations for rheological characterization of hydrogels [42], small-amplitude oscillatory shear tests were performed (AR-G2 Rheometer, TA Instruments) using a 20-mm-diameter parallel plate geometry and hydration chamber to mitigate evaporation. 150 µL acellular hydrogel solution was dispensed between plates. Parameters were maintained across hydrogel formulations, except for temperature and equilibrium time. After shear rheometric parameters were set (Table 1), the plate was rapidly heated or cooled to begin gelation. Agarose-containing hydrogels were tested at a temperature of 23 °C, as these hydrogel formulations were able to complete gelation at this temperature, whereas collagen-only hydrogels were maintained at 37 °C for the test duration. Equilibrium time parameters were also based on published recommendation [42] for agarose-only and collagen-only samples. Composite equilibrium times, meanwhile, were set to match the collagen-only equilibrium time, as the collagen component was expected to be more sensitive to storage ($G{\prime})$ and loss (${G}^{{\prime}{\prime}}$) moduli were defined as the average of all values recorded per step.

**Table 1:** Parameters for shear rheometric testing

	Frequency sweep			Strain sweep			Time sweep		
	4% Agarose	Agarose–collagen composites	4 mg/mL collagen	4% agarose	Agarose–collagen composites	4 mg/mL collagen	4% agarose	Agarose–collagen composites	4 mg/mL collagen
Temperature (°C)	23	23	37	23	23	37	23	23	37
Gap size (μm)	1000	1000	1000	1000	1000	1000	1000	1000	1000
Equilibrium time (sec)	30	60	60	30	60	60	–	–	–
Frequency (Hz)	0.01–100	0.01–100	0.01–100	1	1	1	1	1	1
% Strain	10	10	10	0.1–100	0.1–100	0.1–100	10	10	10

A frequency sweep between 0.01 and 100 Hz was used to evaluate the crosslinking behavior of the hydrogels. Strain sweeps from 0.1 to 100% strain were used to assess the linear viscoelastic region (LVR) limits on fully formed gels. A time sweep was used to confirm consistent mechanical behavior over time and that no unexpected increase in loss or storage modulus would occur due to innate material properties during culture. Each sample (*n* = 3) was only used for one sweep.

### Unconfined Compression of Hydrogels

The effect of agarose and collagen concentrations on equilibrium moduli was evaluated by uniaxial unconfined compression tests. Bulk mechanical changes were assessed in both acellular (*n* = 6; after 24 h gelation) and cell-laden hydrogels (*n* = 4; throughout 21 days of culture) using the same protocol. First, the diameter and heights per each individual sample were measured with a digital caliper prior to testing on a universal testing machine (ElectroForce 5500, TA Instruments) equipped with a 20-lbf load cell and then maintained in a 1 × PBS bath during tests. Stress relaxation was then performed to 10% strain and held (acellular: 1200 sec; cell-laden: 600 sec) to evaluate the compressive modulus and time-dependent behavior (10% strain, 1% strain/sec). The load vs time data obtained from these tests were converted to stress vs time data by applying sample geometry information. These data were then used to estimate the viscoelastic properties of the hydrogels using a nonlinear Prony Series model [43]:

1 $$\sigma \left(t\right)={\sigma }_{\infty }+{\sigma }_{1}{e}^{-\frac{t}{{\tau }_{1}}}+{\sigma }_{2}{e}^{-\frac{t}{{\tau }_{2}}},$$

where ${\sigma }_{i}$ and ${\tau }_{i}$ are stress parameters and relaxation time constants, respectively. From these outputs, the equilibrium modulus (${E}_{\infty }={\sigma }_{\infty }/\varepsilon$) and instantaneous modulus (${E}_{0}=\left({\sigma }_{\infty }+{\sigma }_{1}+{\sigma }_{2}\right)/\varepsilon$) could be calculated by normalizing the experienced stresses to the applied strain [44]. The model was fitted to experimental data using a nonlinear least squares method in MATLAB (R2022a, MathWorks).

### Electron Microscopy of Acellular Hydrogels

Texture and homogeneity of hydrogels (*n* = 3) were analyzed by field emission scanning electron microscopy (FE-SEM; SEM). Samples were flash frozen in liquid nitrogen, fractured with a frozen razor blade, and stored at − 80 °C. Hydrogels were lyophilized (VirTis, SP Scientific) for 48 h at − 20 °C followed by 10 h at 20 °C. Samples were sputter coated with 24 nm of Au-Pd (SPI Supplies) and then imaged using a cold field emission high-resolution scanning electron microscope (S-4800, Hitachi) at an operating voltage of 10 kV. SEM images at 100×magnification were used to quantify porosity using an adapted open-source MATLAB script [45], while 10,000 × SEM images were used to quantify collagen fiber diameters using an adapted open-source Fiber Diameter Distribution v1.0.3 script [46, 47]. In short, to quantify porosities, the image segmentation code segmented images using adaptive thresholding, relying on the mean intensity of a local neighborhood rather than global histogram-based thresholding (i.e., Otsu’s method). An iterative refinement process was applied through erosion and dilation to enhance the segmentation of the region. However, due to the distinct variations between the collagen fibers and background in the 10,000 × images, mean and Gaussian filters were applied. Subsequently, column and row sweeping operations were carried out. Additionally, 2000 × SEM images were used for qualitative comparison. While the porosities of these hydrogels should not be directly linked to structural gaps through which cells might traverse due to ice crystals formation influenced by lyophilization variables (i.e., freezing method, time, and sublimation variables) [48, 49], it is possible to examine the interconnection of hydrogel networks by considering how their crosslinking properties influence the formation of pore artifacts and therefore their effect on cell migration [48, 50].

### Cell Growth

As a surrogate to evaluate cell viability and proliferation, a resazurin assay was used at the initial time point (immediately following cell-laden hydrogel gelation) and intermittently over 21 days of culture. Hydrogels (*n* = 3) were incubated at 37 °C, 5% CO_2_ for 4 h in a solution of DMEM supplemented with 10% resazurin dye following the manufacturer’s protocol (AR002, R&D Systems). Fluorescence was quantified using an excitation of 530/15 nm and emission of 590/15 nm on a fluorescent plate reader (BioTek Cytation 5, Agilent Technologies).

### Extracellular Matrix Production

The sGAG content in cell-laden hydrogel constructs was measured to compare ECM remodeling with respect to hydrogel formulations. After 3, 7, 14, or 21 days of culture, hydrogels (*n* = 3) were weighed, flash frozen, lyophilized overnight, and reweighed. Hydrogels were digested in 1 mL of 50 µg/mL Proteinase K (P6556, Sigma Aldrich) in 50 mM Tris, 1 mM CaCl_2_, pH = 8 for 16 h at 56 °C followed by 30 min at 90 °C and an additional digest of 4 units of beta-agarase (M0392S, New England BioLabs) for 1 h at 65 °C to ensure full agarose breakdown. Supernatants were collected for dimethylmethylene blue (DMMB) assays, and chondroitin sulfate from bovine trachea (C9819, Sigma Aldrich) was used as a standard. DMMB solution was added (200 µL/well) and absorbance was measured at 540 nm and 590 nm using a plate reader (BioTek Cytation 5, Agilent Technologies). For all samples, sGAG quantity was normalized against hydrogel dry weight.

### Statistical Analysis

Results are reported as mean ± standard error (SE) for tests using at least three replicates. Preliminary studies on acellular hydrogels for compression testing, and a 1-week culture of cell-laden hydrogels for resazurin and sGAG assays, showed that 3 samples were sufficient to achieve a power of 0.8. Two-sided, unpaired t-tests were used in rheometry analyses to detect differences in hydrogel plateau or transition points, in SEM imaging to detect pore diameter differences, and in fluorescence imaging to detect nuclear morphology differences. One-way ANOVA with post-hoc Bonferroni or Tukey corrections was used to detect temporal influences in addition to hydrogel formulation influences during compression tests and sGAG tests. *p-*Values less than 0.05 were considered statistically significant. Statistical analyses were performed with MATLAB (R2022a, MathWorks).

## Results

### Agarose Dominates the Mechanical Characteristics of Composite Hydrogels

**Fig. 1:**
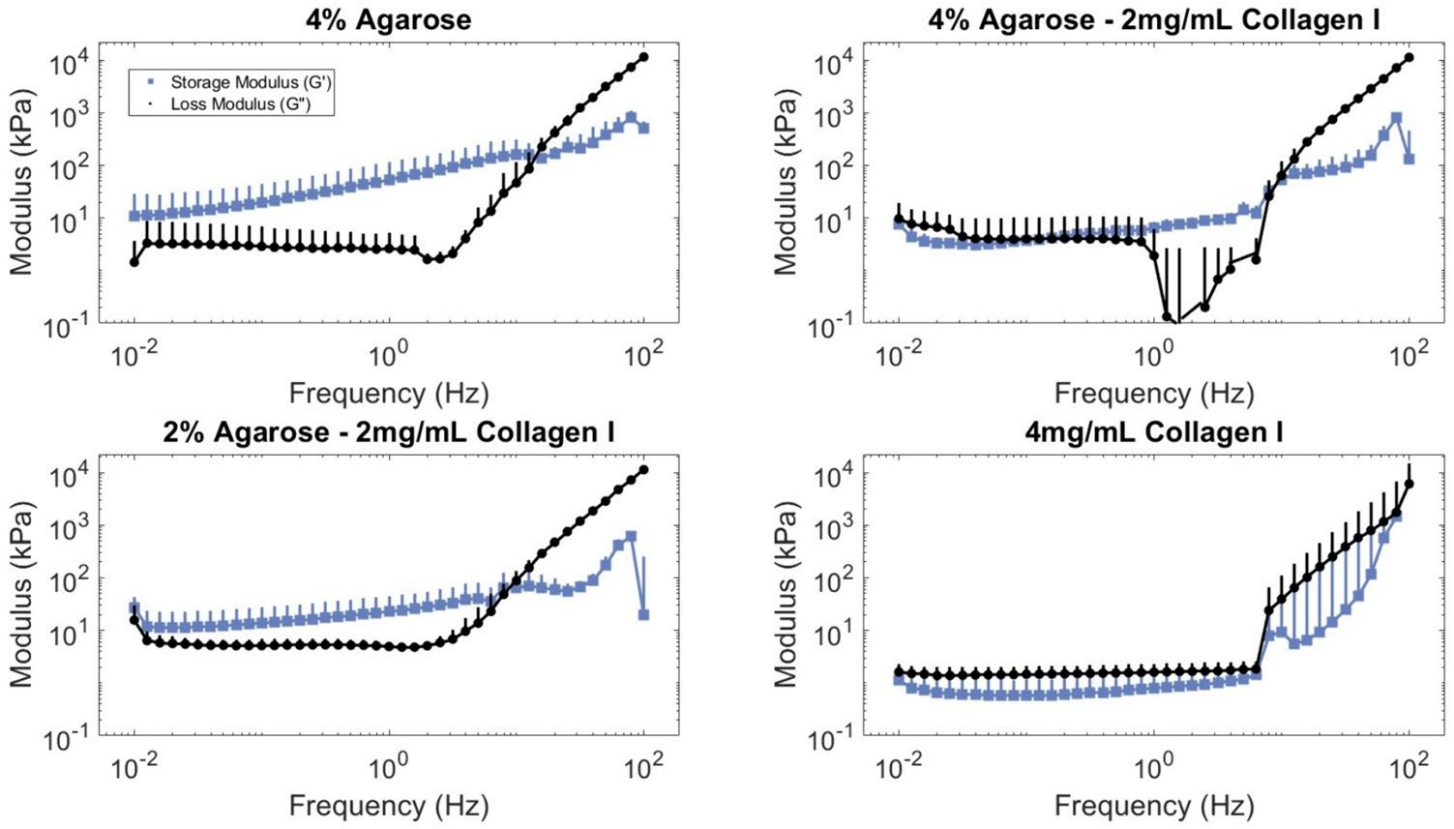
Addition of collagen to agarose reduced the frequency at which the storage and loss moduli cross. The linear equilibrium modulus is defined at the plateau region of $G{\prime}$, which occurs at low frequencies for all pipetted hydrogel formulations. $G{\prime}$ and $G{\prime}{\prime}$ are shown between 0.01 and 100 Hz and zoomed into the region between 1 and 10 Hz for 4% agarose, 4% agarose–2 mg/mL collagen, 2% agarose–2 mg/mL collagen, and 4 mg/mL collagen hydrogels. Three replicates presented as mean + SE

All hydrogels exhibited sol–gel transition, as evidenced by a crossover point during frequency-sweep tests (Fig. 1). 4 mg/mL collagen hydrogels reach the sol–gel transition at the lowest frequency (6.07 ± 1.48 Hz), followed by the two intermediate composites (2% agarose–2 mg/mL collagen: 8.37 ± 1.74 Hz and 4% agarose–2 mg/mL collagen: 10.18 ± 1.34 Hz) and then 4% agarose hydrogels (12.81 ± 1.69 Hz). At frequencies above 10 Hz (10–100 Hz), variations in sample responses increase rapidly. The sol–gel transition is a point along the frequency response curve to an oscillatory input at which the loss moduli become higher than the storage moduli, indicating the samples becomes highly deformable, or more liquid-like than solid-like [51]. The relationship between the storage and loss moduli trends indicated that at low frequencies, the samples exhibit gel-like behavior whereas at higher frequencies, samples begin to display viscoelastic-solid-like properties.

**Fig. 2:**
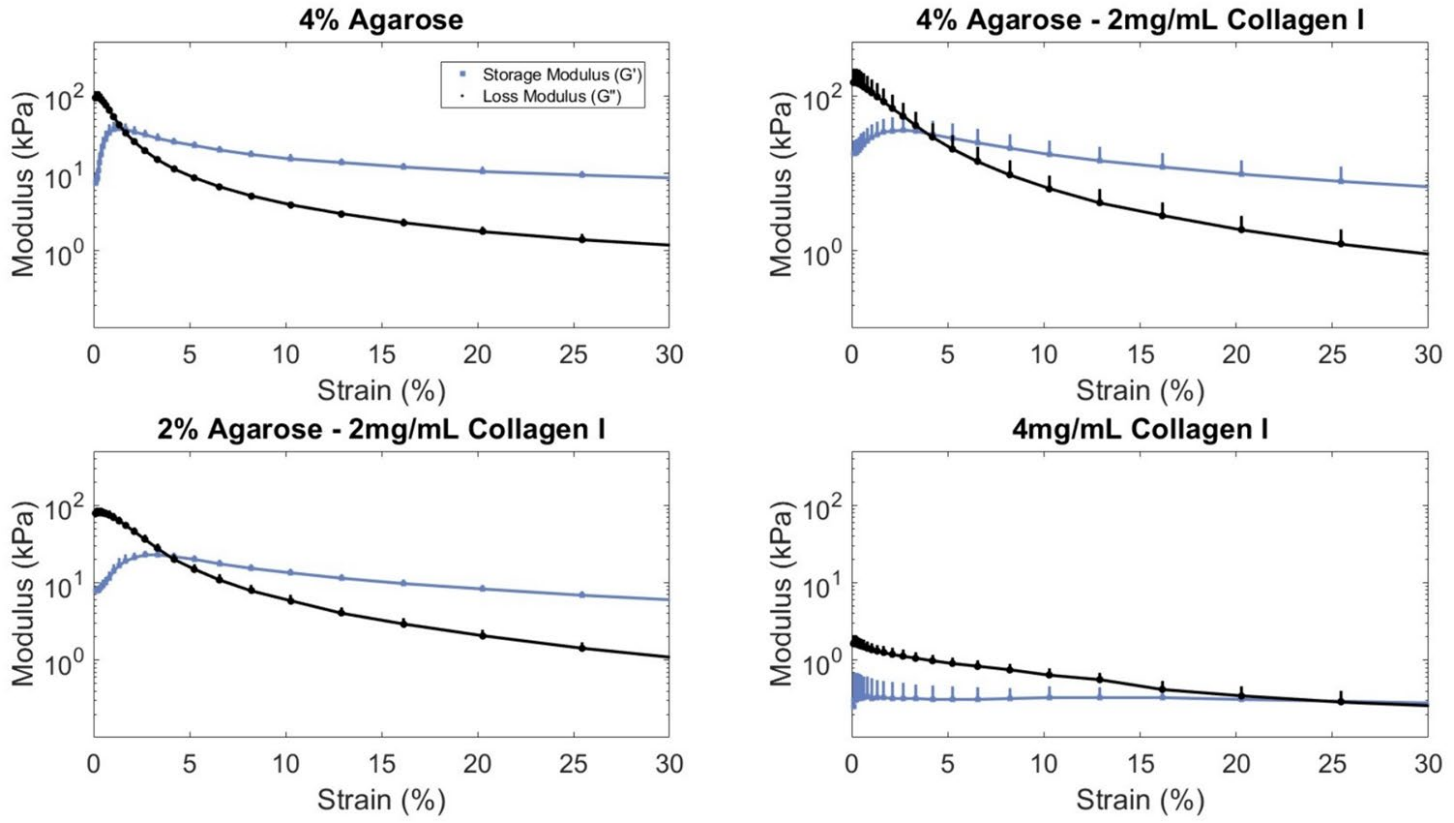
Agarose-only and composite agarose–collagen hydrogels achieved the gel–sol transition point at similar strains. The linear viscoelastic region can be determined with respect to strain where $G{\prime}$ and $G{\prime}{\prime}$ are calculated between 0.1 and 100% strain for all pipetted hydrogel formulations. Three replicates presented as mean + SE

All hydrogels displayed a plateau in moduli by 30% strain during strain sweeps (Fig. 2). The agarose-only hydrogel required the least strain (1.34 ± 0.32% strain) to achieve the gel-sol transition. Composite 4% agarose–2 mg/mL collagen and 2% agarose–2 mg/mL collagen hydrogels achieve this transition point at a higher but insignificantly different strain (4.82 ± 1.39% and 4.72 ± 0.74% strain, respectively). The collagen-only hydrogel on the other hand is more compliant compared to agarose hydrogels and reached the gel-sol transition point at a significantly (*p* < 0.05) higher strain amplitude (26.8 ± 9.53%).

**Fig. 3:**
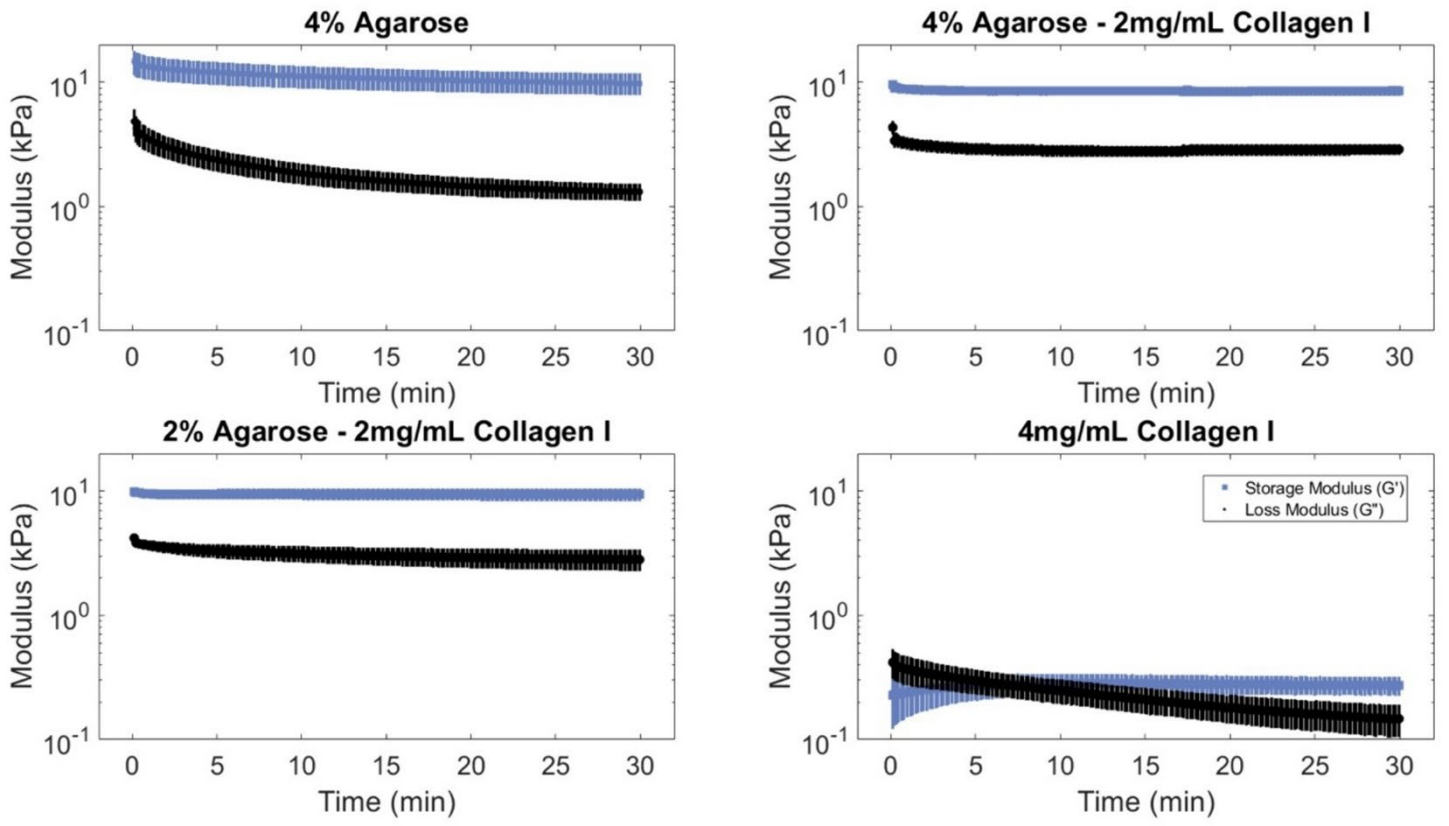
All hydrogel formulations demonstrated stabilized mechanical behavior within 30 min. Gel formation was monitored for 30 min for all hydrogels. $G{\prime}$ and $G{\prime}{\prime}$ are presented for all formulations. Three replicates presented as mean + SE

Within the first 30 min of rheometric time sweeps, all hydrogels reached a plateau, indicating that the hydrogels should maintain their mechanical behavior for sustained culture (Fig. 3). A storage modulus greater than the loss modulus indicates solid behavior, of which all agarose-containing hydrogels clearly demonstrate (*p* < 0.001) before collagen-only hydrogels achieve a similar gelled state, though at an order of magnitude softer.

**Fig. 4:**
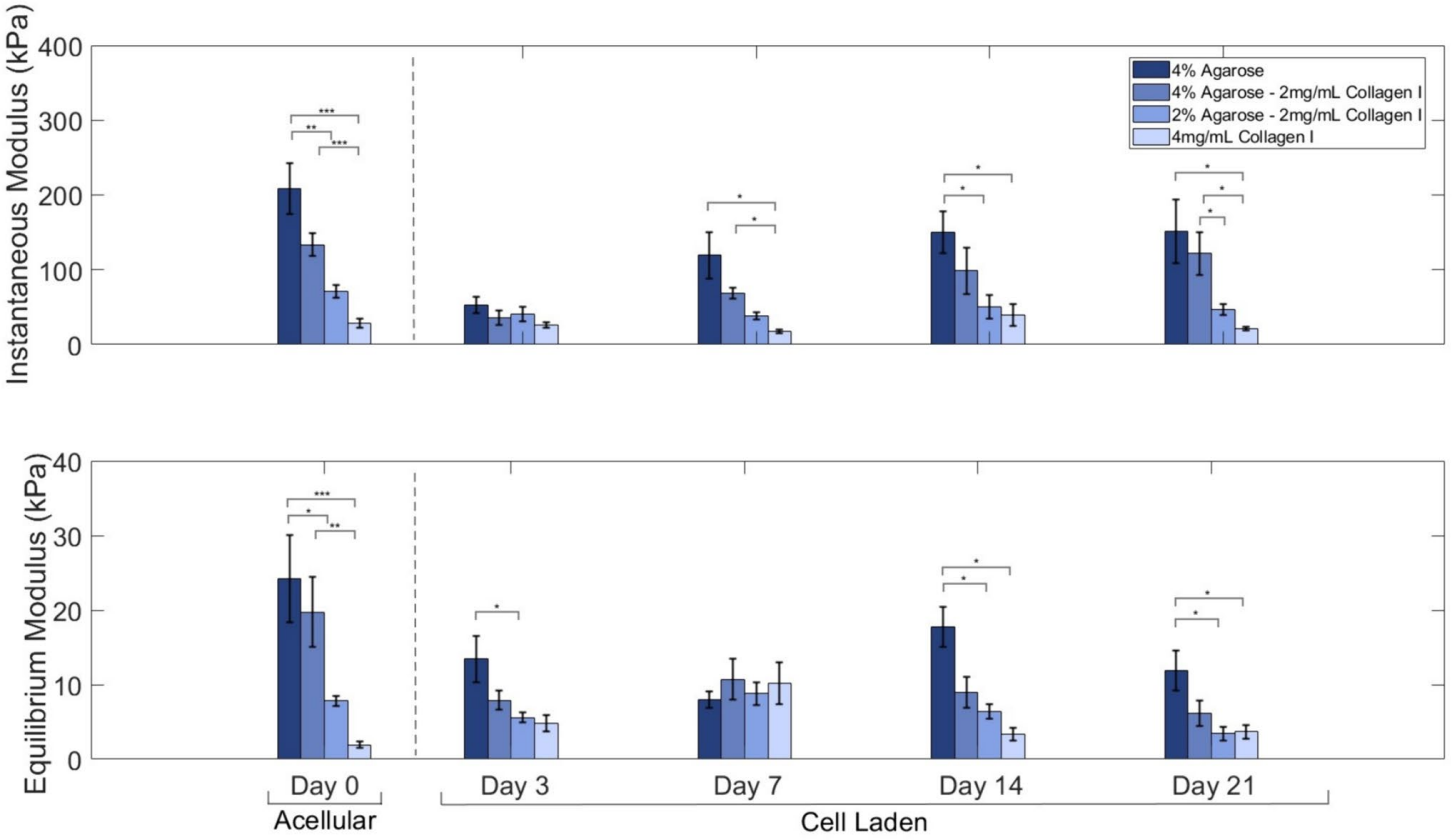
Agarose-only and 4% agarose–2 mg/mL collagen hydrogels displayed similar moduli. Fully gelled hydrogels (*n* = 6 for 24 h acellular studies and *n* = 4 for 21 day cell-laden studies) were tested under unconfined, uniaxial compression-based stress relaxation in a randomized order, compared using a one-way ANOVA with post-hoc Bonferroni correction (*α* = 0.05) and presented as mean ± SE

In acellular hydrogels, equilibrium modulus was the main interest, as this metric best represents the stiffness of the hydrogels in a swollen state, the same state these samples will undergo during later cell-laden experiments. Stress relaxation under unconfined compression (Fig. 4) demonstrated the 4% agarose hydrogels had a similar equilibrium modulus (24.24 ± 5.83 kPa) to the 4% agarose–2 mg/mL collagen hydrogels (19.72 ± 4.68 kPa). 2% agarose–2 mg/mL collagen displays significantly lower equilibrium moduli (7.86 ± 0.67 kPa) compared to the 4% agarose-containing hydrogels (*p* < 0.05) in addition to the 4mg/mL collagen hydrogels (1.93 ± 0.46 kPa) showing significantly lower levels than both 4% agarose–2 mg/mL collagen (*p* < 0.01) and 4% agarose (*p* < 0.001). Results demonstrate similarities with shear rheometry outputs, as both storage moduli discussed above, and equilibrium moduli here represent the elastic behavior of viscoelastic materials, which are more prominent in the 4% agarose-containing hydrogels.

Cell-laden, agarose-containing hydrogels displayed minimal compaction over time, displaying an average diameter increase of 0.09 mm and height decrease of 0.77 mm from initial dimensions. In comparison, collagen-only hydrogels displayed significant changes (*p* < 0.05) in height and diameter (0.47 ± 0.26 mm and 0.80 ± 0.32 mm, respectively) after 21 days of chondrocyte culture.

Initially, stiffer hydrogels, with respect to the initial equilibrium moduli previously discussed, demonstrate a greater change in instantaneous modulus as compared to softer, collagen-dominant hydrogels. However, an important note is that the exposure to cells and culturing conditions will alter the mechanical behaviors of the hydrogels. Therefore, direct comparisons between acellular and cell-laden hydrogel properties should be interpreted with caution. Except for these collagen-only hydrogels, the instantaneous moduli continually increase over 21 days in culture. The final (day 21) instantaneous modulus for 4% agarose hydrogels is 151.77 ± 42.58 kPa, for 4% agarose–2 mg/mL collagen is 121.77 ± 28.93 kPa, for 2% agarose–2 mg/mL collagen is 47.10 ± 7.32 kPa, and for 4 mg/mL collagen is 21.63 ± 2.21 kPa (Fig. 4).

A similar generalization can be made for the equilibrium moduli over time, though there is much higher variability across time points assessed. Statistically, however, temporal effects were not significant across the 21 days assessed. The introduction and culture of chondrocyte into the hydrogel does cause a decrease in moduli when compared to the initial acellular hydrogels. In fact, after 21 days in culture, the equilibrium moduli all decrease to roughly half of the initial acellular levels, whereas in terms of instantaneous moduli, only agarose-containing hydrogels demonstrate a decrease, both of which may be attributed to cellular remodeling of the matrix. The final (day 21) equilibrium modulus for 4% agarose hydrogels is 11.87 ± 2.68 kPa, for 4% agarose–2mg/mL collagen is 6.11 ± 1.70 kPa, for 2% agarose–2 mg/mL collagen is 3.45 ± 0.91 kPa, and for 4 mg/mL collagen is 3.67 ± 0.94 kPa (Fig. 4).

### Higher Agarose Concentrations are Associated with Larger Pore Diameters

**Fig. 5:**
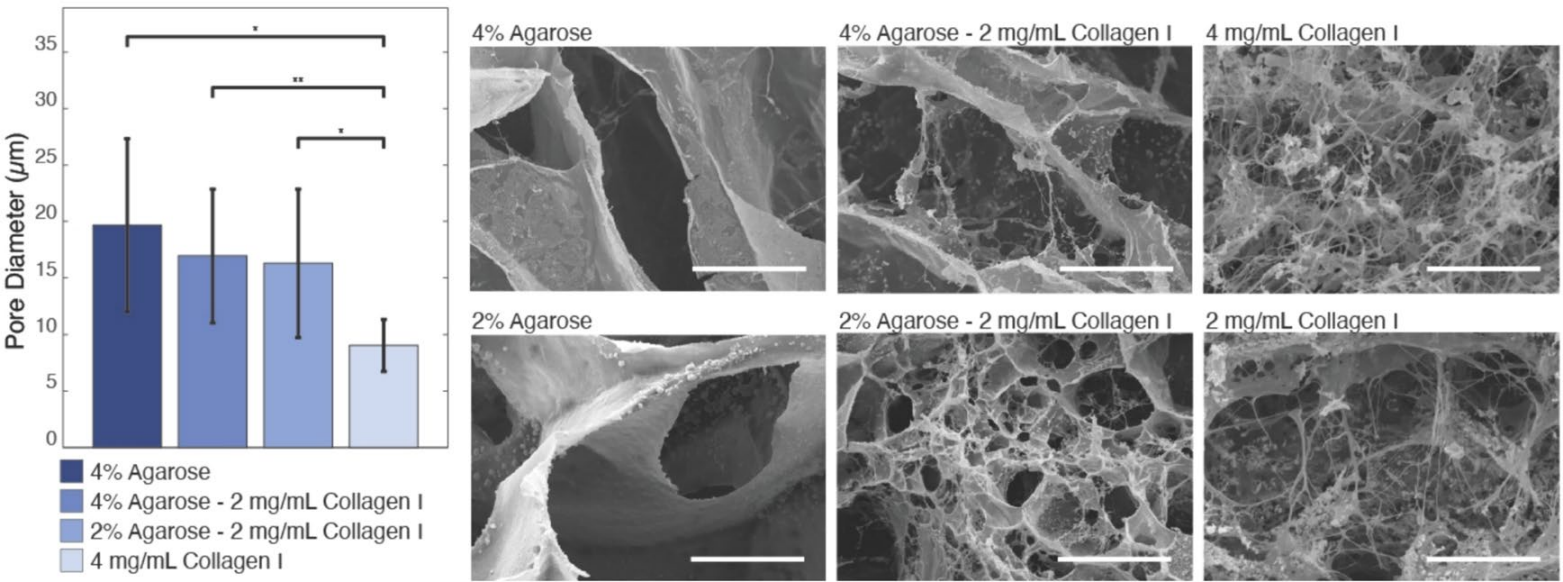
Addition of collagen to agarose reduced pore diameters in SEM images. Pore size was quantified using 3 biological replicate 100 × images of lyophilized hydrogels and assessed using a one-way ANOVA with post-hoc Bonferroni correction (*α* = 0.05), presented as mean ± SE. 2000 × representative images of hydrogels demonstrate fiber presence and morphology. Scale bars = 20 μm

Hydrogel pore diameters were compared at 100×magnification via SEM (Fig. 5). The largest pore diameters were found in 4% agarose hydrogels (19.57 ± 7.61 μm) followed by 2% agarose hydrogels (19.044 ± 7.33 μm), 4% agarose–2mg/mL collagen, and 2% agarose–2 mg/mL collagen pore diameters (16.90 ± 5.90 μm and 16.21 ± 6.55 μm, respectively). Significantly smaller (*p* < 0.05) pore diameters were observed in 4 and 2 mg/mL collagen samples (9.10 ± 2.68 μm and 12.48 ± 4.10 μm, respectively). Agarose-only hydrogels demonstrated a positive correlation between concentration levels and resultant pore diameters, whereas collagen-only hydrogels demonstrated a negative correlation of pore and fiber diameter at 100× and 10,000× magnification levels, respectively.

### Composite Hydrogels Display Intermediate Cell Responses between Agarose- or Collagen-only Hydrogels

**Fig. 6:**
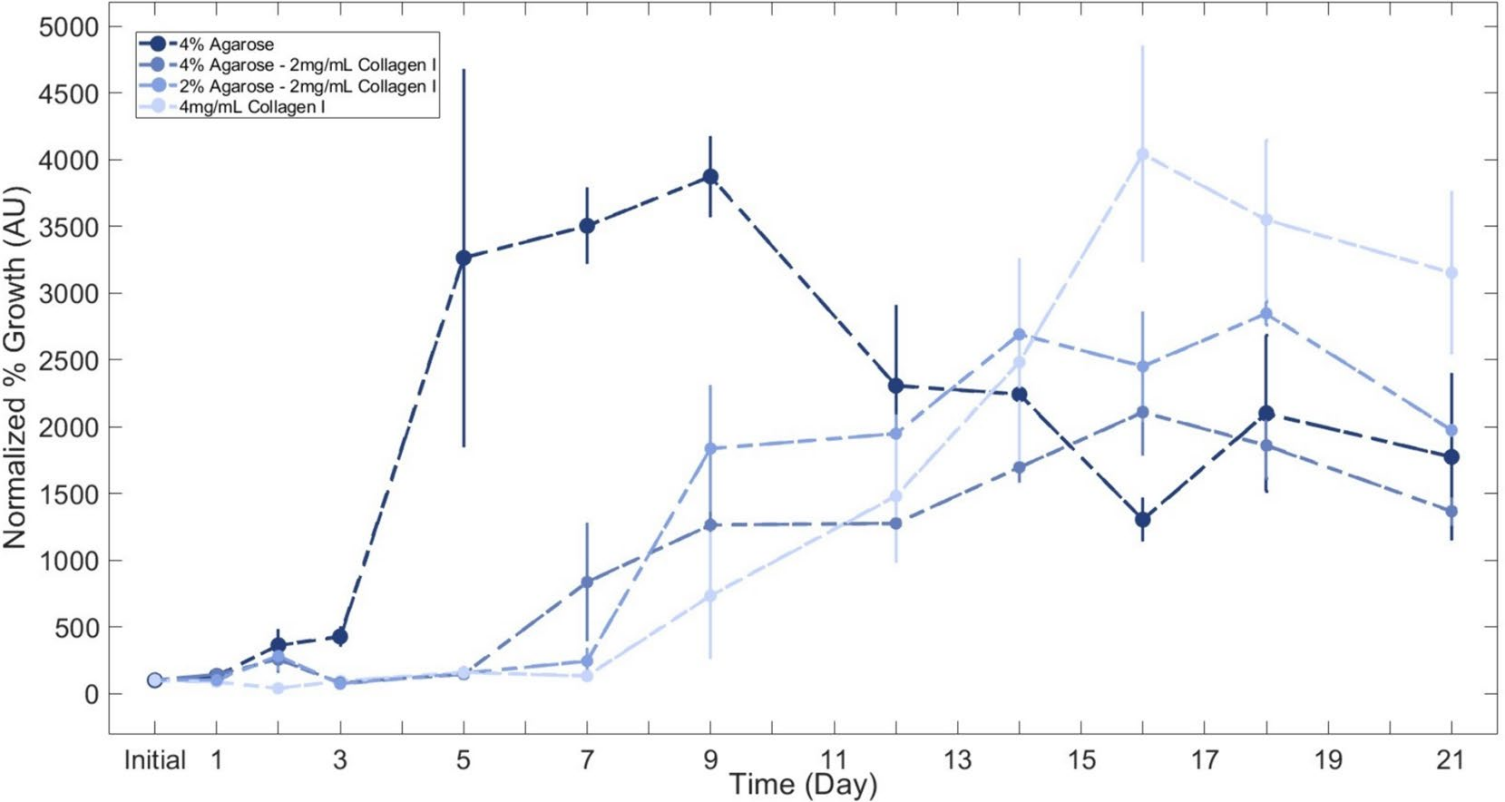
Chondrocyte metabolic activity, an indicator of cell viability and proliferation, demonstrated cell growth across all hydrogels. Cell viability is presented as a percentage of baseline (100%) levels, using 3 biological replicates and presented as mean ± SE

Since cells were briefly subjected to mechanical and thermal stress during hydrogel formulation methods, cell viability was initially measured directly following cell seeding to establish a baseline. All hydrogels maintained the seeded chondrocytes within their 3-dimensional matrix and demonstrated continued growth, as assessed via resazurin assays and visual inspection under brightfield microscopy. Agarose-only hydrogels demonstrated the quickest initial increase in growth; collagen-only hydrogels initially lagged but eventually demonstrated the largest growth rates (Fig. 6). Composite hydrogels meanwhile displayed similar rates to each other and followed the progression cell growth curves through the lag, log, and stationary phases (Fig. 6).

### Hydrogel Formulation is not a Significant Driver in Observed sGAG Content

**Fig. 7:**
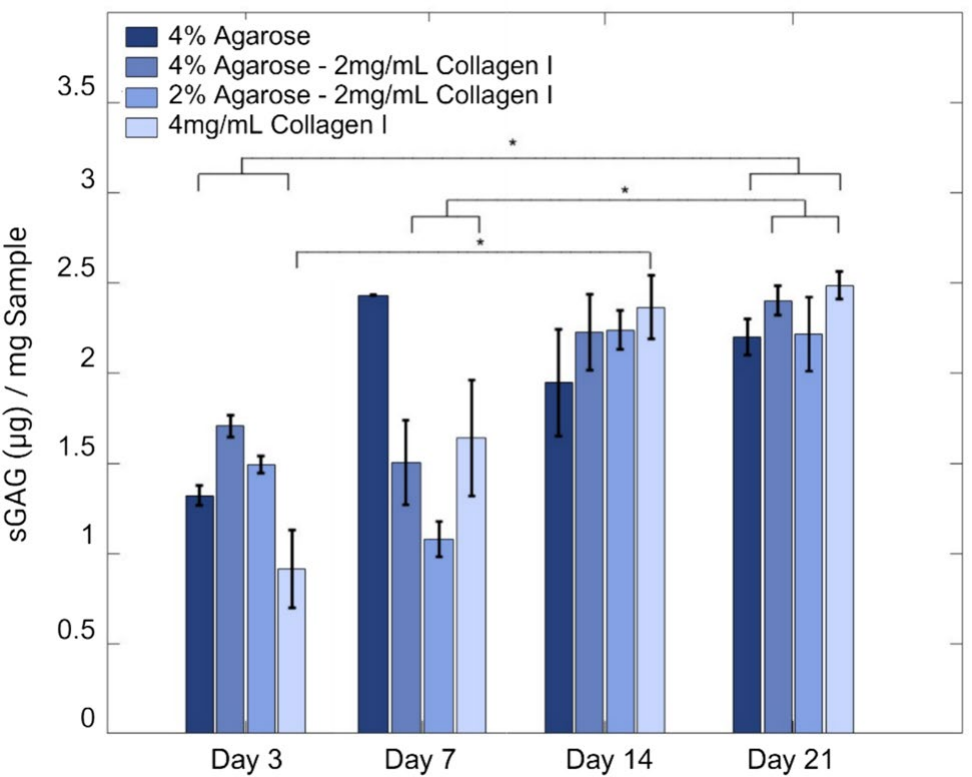
Collagen-containing hydrogels demonstrated an increase in sGAG content that plateaued by day 21. One-way ANOVA with post-hoc Bonferroni correction of multiple pairwise comparisons (*α* = 0.05) was used to detect temporal and hydrogel formulation influences on sGAG content in hydrogels (*n* = 3) and results are presented as mean ± SE

sGAG was analyzed normal to sample dry weight. Interestingly, both composite hydrogels demonstrated a brief decrease in sGAG content between days 3 and 7 before continuing to show continuous increases in content. DMMB results demonstrate on day 3, and sGAG content in 4% agarose–2 mg/mL collagen hydrogels was statistically higher than other groups (Fig. 7). Other than this initial case, however, the temporal influence was the main driver on sGAG content (*p* < 0.01), while formulation alone was not a significant driver (*p* = 0.7), indicating that the addition of agarose in the composite hydrogels does not hinder the ability of chondrocytes to secrete sGAG in vitro at the levels tested.

## Discussion

Biomaterials meeting the mechanical and biochemical requirements for functional environments during cell culture are necessary for mechanotransduction. This study aimed to evaluate agarose–collagen composite hydrogels as a simple, effective option for chondrocyte mechanobiology studies. Four hydrogel formulations were assessed, including a high-concentration, 4% agarose hydrogel, two common-level collagen concentrations with varied agarose (4% agarose–2 mg/mL collagen and 2% agarose–2 mg/mL collagen), and a 4 mg/mL collagen hydrogel. Both acellular and prolonged cell-laden studies indicate that high-concentration agarose blended with collagen hydrogels may be a suitable environment for extended, 3-dimensional chondrocyte culture.

### Mechanical Properties Describe the Hydrogel Structure–Function Relationship

Of the sweeps performed, all acellular hydrogels demonstrated linear viscoelastic characteristics that fall within the bounds of common parameters used in loading studies [42, 52], a critical factor when developing a hydrogel material meant for culture in physiologically relevant, dynamic conditions. The addition of collagen into agarose did not significantly change the rheologic properties of the hydrogel though it did slightly reduce the storage modulus. Similar to Cambria et al. [32], we did not observe a significant difference between the moduli of agarose-only and 4% agarose–2 mg/mL collagen I (both at roughly 10 kPa) and as the agarose content decreases, so do the moduli, with a particularly sharp drop between composite hydrogels and collagen-only hydrogels. The blending of collagen and agarose at these concentrations therefore does not seem to impair bond formation during gelation, though it is possible that at higher collagen concentrations, fiber aggregation might interfere with gelation, leading to macroscopic defects, as suggested by the results found by Cambria et al. [32], and SEM images of collagen-only samples.

To assess how the addition of collagen may influence the compressive properties of agarose hydrogels, unconfined, uniaxial compression testing was completed. Acellular 4% agarose hydrogels were the stiffest material after gelation, followed by 4% agarose–2 mg/mL collagen, which both fall within the lower bounds of native cartilage PCM [19, 20]. Acellular agarose-containing hydrogels have been previously reported to express significantly higher equilibrium moduli as compared to cell-laden hydrogels of the same formulation due to the presence of cells forming an extracellular matrix [53]. The moduli values of the composite hydrogels demonstrate a dependence on the volume fractions of the agarose and collagen, in which hydrogels with higher agarose content showed improved mechanical properties, reflecting changes to hydrogel moduli reported in other studies. 0.5% agarose hydrogels have an equilibrium modulus ranging from 5 to 8 kPa [33], 2% agarose hydrogels at 14 kPa [53], 3% agarose hydrogels at roughly 20 kPa [14], and 4% agarose hydrogels at 40 kPa [53]. Low-concentration agarose–collagen hydrogels have been previously shown to demonstrate a spread in behavior over a week of culture similar to the behavior of the 2% agarose–2 mg/mL collagen hydrogels [33]. Meanwhile, collagen-only hydrogels demonstrated substantially lower equilibrium moduli following compression [54], like that of 2.3 mg/mL collagen hydrogels, which have a modulus of only 0.1 kPa [29]. Cell-laden agarose-containing hydrogels presented the most structural stability over time, in part due to the lack of agarose production by chondrocytes. Since the cells do not produce any enzymes that break down agarose, the material does not erode quickly [34]. Collagen-based hydrogels, on the other hand, are well known to contract over time, decreasing upward of 70% in diameter [23, 28]. Supplementing observations from rheologic testing, the low levels of contraction observed in the composite formulations demonstrated how the agarose component dominated over collagen to maintain the hydrogel shape with time. This presents an advantage for studies in which hydrogels need to be cultured for long durations or undergo loading to mimic physiologic conditions.

In general, the relaxation behavior of the hydrogels seems to be dominated by the agarose composition. The observed increase in moduli over time may be due to greater fiber engagement or increased matrix deposition as the cells continue to proliferate [35]. In prior studies, acellular agarose hydrogels had an equilibrium modulus of 17.7 ± 2.7 kPa after a week of culture [14], and 2% agarose hydrogels had an equilibrium modulus of 14.2 ± 2.7 kPa after gelation [53]. On the other hand, collagen-based hydrogels show almost an order of magnitude lower modulus. For example, equilibrium modulus fell in the 0.5 kPa range, with a higher instantaneous modulus of roughly 5 kPa, at the same time point [29]. The equilibrium moduli range we measured in 4% agarose and 4% agarose–2 mg/mL collagen I after full gelation (19.72 ± 4.68 kPa) fell within previously reported ranges for agarose [14, 53].

We observed that increasing agarose content results in stiffer hydrogels but that the addition of cells affects the overall modulus as a function of culture time. These trends in material properties parallel those observed by others. Buckley et al. showed that modulus increased twofold for every 2% agarose increase within the 2–6% range [53]. In cell-laden hydrogels, Buschmann et al. found a slow increase in stiffness over the first 2 weeks of culture [14]. By day 28, the modulus was about 80 kPa, before dropping back to roughly 65 kPa by day 47 [14]. In addition, our acellular hydrogels maintained an equilibrium modulus consistently around 17 kPa, indicating that matrix deposition by resident cells plays a crucial role in the structural integrity of the hydrogel. Buckley et al. additionally noted that parameters related to the experimental methods (i.e., batch-to-batch variability, cell seeding density) influenced the equilibrium modulus; they demonstrated several cases where acellular hydrogels displayed a significantly higher modulus than those hydrogels seeded with a high concentration of cells [53]. Congruent to these findings, over the 21 days of culture in our study, cell-laden hydrogels demonstrated lower moduli than acellular hydrogels immediately following gelation.

Pore diameter sizes seem to follow the same trend as equilibrium moduli, in which 4% agarose hydrogels, the material with the largest pore diameter, also demonstrate the highest moduli. This related change in pore geometry and subsequent material property are likely attributable to the reduction of hydrogel bonding with decreased agarose concentrations [30, 33, 55, 56]. The composite hydrogels demonstrate similar porosities and moduli but are both lower than agarose-only hydrogels. These composites are double networked since they consist of contrasting component properties and molar concentrations. The differences in crosslinking mechanisms between materials within these hydrogels likely influence the observed trends in pore and modulus values [57–59]. Both agarose and collagen are capable of hydrogen bonding [60], suggesting the potential for crosslinking. This is supported by the porosity changes we observed from SEM imaging, whereby decreasing the concentration of agarose relative to collagen resulted in reduced porosity and smaller pore sizes. Such crosslinking was observed by Quarta et al. [33], and the homogeneity of such networks also demonstrated by others [32]. Cell adhesion and contractility have been previously studied in 0.5 mg/mL collagen hydrogels with 0–0.5% w/v agarose to demonstrate agarose-mediated inhibition of collagen fiber bundling, deformation, and remodeling [34]. The group also discovered that by covalently crosslinking the hydrogel with glutaraldehyde, cells were able to spread more effectively with minimal matrix remodeling, indicating that agarose alone does not limit cell spreading [34]. Furthermore, by increasing collagen concentrations, therefore increasing ligand density, they were also able to overcome cell spreading limitations. This points to the possibility of initial covalent crosslinking between hydrogels being increased at our higher concentration. These connections across polymer materials potentially benefit cell–matrix interactions by providing cells initial binding sites on which to further deposit extracellular matrix proteins [61].

Collagen-only hydrogels, meanwhile, demonstrate a negative relationship between porosity and moduli, possibly mediated in part by bond strengths, as there exists a positive relationship between collagen concentration and ionic strength [56] and further a positive relationship between increases in ionic bond strength and fiber network connectivity [34, 62], leading to smaller pore and fiber diameters. Furthermore, variability between samples can arise and is demonstrated in literature depending on collagen source, polymerization temperature, and pH, all of which affect crosslinking and cellular interactions [48, 49, 55, 63].

### Cell and Extracellular Matrix Responses Describe the Hydrogel Biofunctionality

In this study, the stiffest hydrogel, 4% agarose-only, led to the quickest and most dramatic increase in proliferation on day 7, as measured through mitochondrial activity, although the 4 mg/mL collagen-only hydrogels reached similar growth rates within 2 weeks of culture. Previous reports are inconclusive toward the relationship between hydrogel viscosity and cell proliferation over a 21-day period. For example, Lee et al. found that higher viscosities led to higher proliferation rates [64], whereas Cambria et al. found the opposite [32]. Potential explanations for the contradictory results may be the influence of contact inhibition or biochemical signaling differences across biomaterials (gelatin blends versus agarose–collagen blends, respectively). Since mammalian cells will not bind to agarose polysaccharides, it is possible that the embedded cells were able to begin proliferating much sooner in a free-floating state as compared to the collagen-containing hydrogels that allowed the cells to take time and bind to fibers prior to entering the exponential growth phase. The composite hydrogels resulted in growth curves with peaks between days 14 and 18. Collagen hydrogels achieve peaks in proliferation between days 16 and 21.

No significant differences in sGAG content by time point were observed among the formulations after day 3. Over the course of the study, all hydrogels continued to demonstrate greater sGAG content, suggesting continual matrix synthesis. A lack of consensus exists regarding sGAG content in composite hydrogels. While some reports show higher collagen content associated with higher sGAG content [32], others report the opposite effect, in which a low collagen content led to higher sGAG content [31]. However, as previously referred to, these differences could also be attributable to discrepancies among collagen sources and associated influences on cell behavior.

This study focused on the structural and biofunctional properties of agarose–collagen composite hydrogels. Consistent results for material properties of the hydrogel formulations (i.e., rheologic sweeps, compression testing, contraction) enabled sufficient statistical power, even with only 3 samples. Although the greater variability of biologic analyses (i.e., resazurin, sGAG assays) resulted in reduced power in post-hoc evaluation, our results still demonstrate biocompatibility of these hydrogels for extended cell culture. To gain a deeper insight into the influence of these physical cues of the composite hydrogels, future work should address how cell types interact differently with the hydrogel over time using additional biochemical and immunohistochemistry analyses. These hydrogels can be easily and quickly produced, mechanical shear and compressive properties show promising behavior as stable, long-term environments, microscopy images demonstrate homogenous and interconnected networks for cell growth, and preliminary cell-laden studies demonstrate continual proliferation, matrix deposition, and maintained morphology. Overall, the 4% agarose–2 mg/mL collagen hydrogel formulation showed potential in the context of chondrocyte mechanobiology studies.

## Citation Diversity

Recent work in scientific fields have identified biases in citation practices such that papers of minority scholars are found under-cited [65]. We recognize this bias and have worked to ensure that we are referencing appropriate papers with fair author inclusion.

## Data Availability

Collected data are made available using a Harvard Dataverse repository.
